# Quantification of the Effect of Saddle Fitting on Rider–Horse Biomechanics Using Inertial Measurement Units †

**DOI:** 10.3390/s25154712

**Published:** 2025-07-30

**Authors:** Blandine Becard, Marie Sapone, Pauline Martin, Sandrine Hanne-Poujade, Alexa Babu, Camille Hébert, Philippe Joly, William Bertucci, Nicolas Houel

**Affiliations:** 1 LIM France, 24300 Nontron, France; msapone@lim-group.com (M.S.); pmartin@lim-group.com (P.M.); shannepoujade@lim-group.com (S.H.-P.); chebert@lim-group.com (C.H.); 2 Laboratoire Performance, Santé, Métrologie, Société, Université de Reims Champagne Ardenne, UFR STAPS, 51100 Reims, France; philippe.joly@univ-reims.fr (P.J.); william.bertucci@univ-reims.fr (W.B.); nicolas.houel@univ-reims.fr (N.H.)

**Keywords:** biomechanics, horse locomotion, rider–horse synchronization, pelvis, saddle fitting

## Abstract

**Highlights:**

**What are the main findings?**

- Inertial sensors offer a reliable alternative for assessing the impact of saddle fitting.
- Saddle fitting adjustments significantly influence horse and rider biomechanics.

**What is the implication of the main findings?**

- Establishing an objective and reproducible method to assess saddle fit is essential.

**Abstract:**

The saddle’s adaptability to the rider–horse pair’s biomechanics is essential for equestrian comfort and performance. However, approaches to dynamic evaluation of saddle fitting are still limited in equestrian conditions. The purpose of this study is to propose a method of quantifying saddle adaptation to the rider–horse pair in motion. Eight rider–horse pairs were tested using four similar saddles with small modifications (seat depth, flap width, and front panel thickness). Seven inertial sensors were attached to the riders and horses to measure the active range of motion of the horses’ forelimbs and hindlimbs, stride duration, active range of motion of the rider’s pelvis, and rider–horse interaction. The results reveal that even small saddle changes affect the pair’s biomechanics. Some saddle configurations limit the limbs’ active range of motion, lengthen strides, or modify the rider’s pelvic motion. The temporal offset between the movements of the horse and the rider changes depending on the saddle modifications. These findings support the effect of fine saddle changes on the locomotion and synchronization of the rider–horse pair. The use of inertial sensors can be a potential way for quantifying the influence of dynamic saddle fitting and optimizing saddle adaptability in stable conditions with saddle fitter constraints.

## 1. Introduction

Horse riding refers to the meeting and association of two living beings, a human and a horse. Their relationship is consequently the key to well-being and performance in equestrian sports [1]. The saddle is an essential component in their connection. The saddle, which is located between the rider and the horse, is a valuable performance tool for the two athletes. To optimize their engagement, the saddle must be fitted for them. Saddle fitting seeks to fit the saddle to the rider and horse. The saddle fitting process typically consists of two steps: a static evaluation and a dynamic evaluation of the rider–horse pair. During the static evaluation, the saddle fitter examines and analyzes the horse’s and rider’s morphology and conformation, as well as gathers information about their practice and takes measurements on the horse standing still. This stage provides initial information for saddle selection. When working, the bodies of the horse and rider behave differently than when they are quietly standing. The horse, for example, will engage its back muscles and change their thickness [2,3]. This is why it is essential to consider the biomechanics of the rider–horse pair while testing saddles in motion to optimize saddle fitting [4]. The dynamic phase of the fitting permits the testing of several saddles and the observation of the rider and horse as they move together.

However, despite the importance of dynamic evaluation, many saddles in practice remain ill-fitted [5]. Moreover, the horse can only express itself physically, so it is necessary to pay attention to its body language. Some horses, however, are less expressive than others and do not exhibit symptoms of distress such as agitation, pinned back ears, or changes in eye expression [6,7]. Furthermore, these observations are subjective, and while they provide indicators, they do not allow for true quantification of saddle fit. In the scientific literature, the method for assessing dynamic saddle fitting is identified as the usual tool used is the pressure sensor mat. Although it has already been validated [8,9], this tool involves a rigorous and meticulous approach [10,11]. According to its specific methodology, pressure sensors have become poorly used by saddle fitters and in equestrian conditions. In order to support saddle fitters in the selection process, new practical methods should be developed to objectively quantify saddle fit. Such methods could also help to improve the harmony of interaction between horse and rider [12]. Tridimensional kinematics techniques are likewise well-known for their precision and reliability [13], but they are also not adapted due to the complexity of their implementation on horses under the required conditions. Nevertheless, 3D kinematics approaches have validated various wearable measurement tools, especially inertial sensors [14,15,16,17]. Inertial sensors have been used to assess rider–horse interaction and tiredness during endurance races [18]. They have also been used to evaluate horse locomotion [19] and rider pelvic range of motion [20]. To our knowledge, although saddle fitting has been investigated in the literature using pressure mats [5,10,21], and while inertial sensor methods have been studied, they have not been tested in order to estimate the influence of fine saddle fitting conditions. The objective of this study is to develop an inertial measurement method to objectively quantify the biomechanical impact of small, predefined saddle modifications on horse–rider kinematics during trotting. We hypothesized that even fine adjustments in saddle fit would significantly alter rider and horse movement patterns and that these effects could be reliably detected using inertial sensors.

## 2. Materials and Methods

### 2.1. Riders and Horses

Eight rider–horse pairs participated in the present study. Each rider–horse pair was in familiar surroundings, and the horses had been in the stables for some months. The riders were 5 women and 3 males, with an average age of 28 ± 8 years (height: 169 ± 7 cm; weight: 64 ± 15 kg). Each rider rode a horse they were accustomed to riding regularly. The horses were six geldings and two mares (age: 12 ± 2 years; height: 166 ± 4 cm). All of the rider–horse pairs were experienced and could easily complete the protocol exercises. They had no health issues or history that could have influenced the outcomes. Each rider gave their informed consent in accordance with the approval of the ethics committee.

### 2.2. Protocol Study

**Figure 1:**
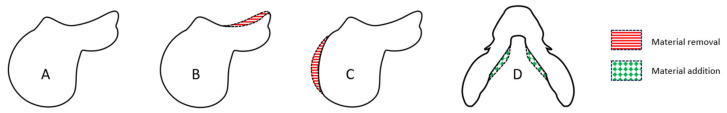
Illustration of the 4 tested saddles.

Two straight lines of at least 20 m were established in the arena and marked with cones. The riders and horses warmed up for 15 min using their personal equipment. Only the saddle was changed for the different conditions following warm-up. The measurements were acquired during the testing of four different saddles at trot. Each pair was instructed to produce straight lines at a rising trot in both clockwise and anticlockwise directions. They were asked to maintain a steady speed. The data from the left- and right-hand recordings were combined to better match the field constraints and the final application of the methods used. Each condition was measured with a minimum of 80 strides (110 average strides per condition). The examined strides were taken when the rider–horse combination was moving in straight lines at steady rate. Four saddles were tested in randomized order (Figure 1). The saddles used in this study were identical models, specifically prepared by a professional saddle fitting workshop to introduce controlled variations for research purposes. The same saddles were used for each pair. One saddle served as the reference configuration (saddle A), and the three others (B, C, and D) each incorporated a single fine-tuning modification while preserving the saddle’s overall structure and integrity. For saddle B, the seat is 6 mm deeper than saddle A to slightly change the rider’s positioning. Saddle C featured a 15 mm reduction in flap width, aiming to modify the rider’s leg contact. Saddle D included a 10 mm elevation of the front panels to change the balance of the saddle. These minor adjustments were performed with precision (industrial accuracy < 1 mm) to isolate the effect of each fitting parameter while maintaining the functional characteristics of the saddles.

### 2.3. Equipment

**Figure 2:**
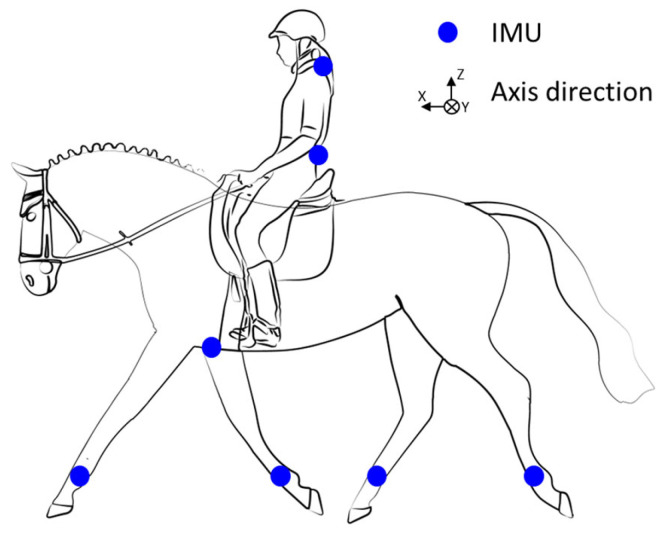
Illustration of the 7 inertial measurement units’ (IMUs’) placement.

The couples were equipped with an Inertial Measurement Units (IMUs) system including seven synchronized IMUs (Blue Trident, dual-g sensors, Vicon Motion Systems Ltd., Oxford, UK) that sampled at 1125 Hz. The IMUs consist of a tri-axial accelerometer (±16 g, precision: 0.00049 g) and a tri-axial gyroscope (±2000°/s, precision: 0.061°/s). Five IMUs were placed on the horse’s four leg cannons and sternum (set up to the girth). The IMUs were synchronized within each acquisition session using Capture.U software (Vicon Motion Systems Ltd., Version 1.3.1), which assigns a common Unix timestamp (µs) to all sensors at recording, ensuring precise temporal alignment throughout the trial [22]. The other two IMUs were attached to the rider (C7 and S1 vertebrae) (Figure 2). Sensor orientations are identical. To reduce speed-related errors, their speed was recorded using a GNSS sensor (Equimètre, Arioneo, Bordeaux, France) (precision 0.18 km/h).

### 2.4. Data Analysis

All data (accelerometer and gyroscope) were filtered using a fourth-order Butterworth type-two low-pass filter with a cutoff frequency of 20 Hz. The properties of these filters were visually picked using the method described by Wells & Winter [23]. The offset of the gyroscopic signals was verified. The variables measured during the four saddle trials included the active range of motion (AROM) of protraction/retraction of the forelimbs and hindlimbs, the horse’s stride time, the pitch AROM of the rider’s pelvis, and the rider–horse synchronization as measured by the time offset. These parameters were examined for all eight couples. The data was processed using Matlab (Matlab R2023b, The MathWorks Inc., Natick, MA, USA).

#### 2.4.1. Active Range of Motion (AROM) Protraction/Retraction (P/R) Fore and Hindlimbs

**Figure 3:**
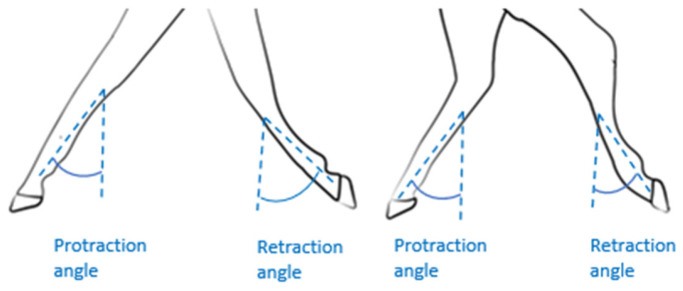
Illustration of the angles of protraction/retraction of the forelimbs and hindlimbs.

Protraction is defined as a limb’s forward extension, while retraction is its backward extension (Figure 3). The amplitude of protraction/retraction is thus calculated as the angular difference between the maximum protraction and maximum retraction during a stride. The approach used was described by Sapone et al. [19]. The angle between the vertical and cannon positions was measured using the gyroscopic signal on the *y*-axis of the IMUs mounted on the horse’s four legs (Figure 2). In this method, the cannon bone is considered to be in a vertical position at the time the withers reach their lowest point [19].

#### 2.4.2. Stride Time (ST)

The stride time is the overall duration of a complete stride or the time it takes for a limb to complete a full cycle from a beginning position to its return point. This metric is essential for understanding the rhythm and cadence of equine locomotion, as well as determining the effectiveness of its motions. The stride time was determined using the gyroscope on the *y*-axis of the IMU of the right forelimb (Figure 2). The signal’s greatest peaks were detected. The time between each peak is calculated to determine the duration of each stride [24].

#### 2.4.3. Rider’s Pelvic AROM

**Figure 4:**
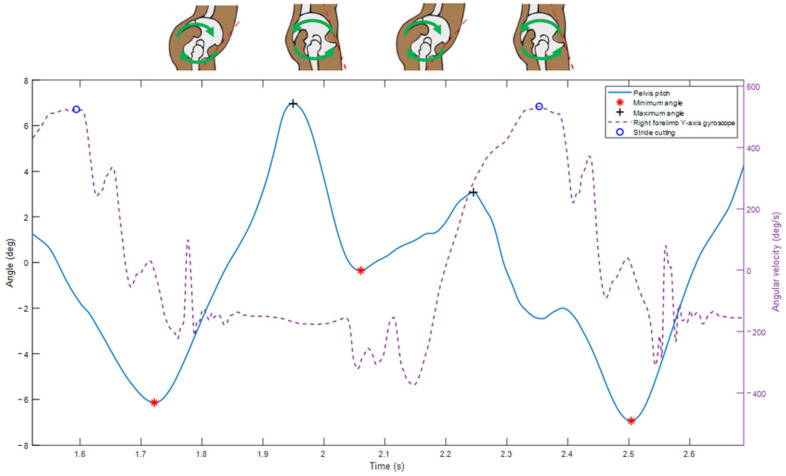
Amplitude of pitch movement of the rider’s pelvis.

The pitch ROM of the rider’s pelvis (Figure 4) was assessed using the method described by Becard et al. [20,25]. The strides were detected using the filtered gyroscopic signal on the medio-lateral axis (*y*-axis) of the IMU mounted on the right forelimb [18]. The AROM of the pelvis was computed using the sensor mounted on the rider’s pelvis (S1 vertebra, Figure 2). The offset of each accelerometer axis was taken into account to initialize the integration of the gyroscope signal. Then, the gyroscopic angular velocity around the sensor’s medial–lateral axis (*y*-axis) was integrated. Drift was minimized using a fourth-order Butterworth high-pass filter type 2 with a cutoff frequency of 1 Hz and order 4. The AROM was calculated as the difference between the pelvis’s minimal and maximum angles when the rider is seated on the saddle.

#### 2.4.4. Rider–Horse Synchronization (Time Lag)

The rider–horse synchronization was measured using the approach described by Viry et al. [18]. This method used displacement signal obtained by the double integration of the filtered acceleration signal from the dorso–ventral axis (*z*-axis) of the horse’s IMU sternum and the cranio–caudal axis (*z*-axis) of the rider’s IMU S1 (Figure 2). The minimum peaks of these two displacement signals are then identified using Matlab (Matlab R2023b, The MathWorks Inc., Natick, MA, USA). The Time Lag (TL) between the minimum position of the rider and that of the horse was finally measured. It reflects the synchronization between horse and rider. The TL is positive when the rider’s movement is delayed relative to the horse and negative when the rider’s movement precedes the horse. A TL of zero corresponds to an absence of measurable phase shift between horse and rider movements.

### 2.5. Statistics

Intra-sample comparisons were made in order to estimate the influence of the saddle on each rider–horse pair. All statistical tests were conducted using MATLAB software, with a significance level of *p* < 0.05. Shapiro–Wilk tests showed that data does not follow normal distributions. Then the Kruskal–Wallis test is used to assess if the distribution of at least one of the saddles differs from the others. If the Kruskal–Wallis test shows a *p*-value < 0.05, for each horse rider pair, saddles B, C, and D were compared to saddle A (reference saddle) using a Wilcoxon–Mann–Whitney test (U-test) (*p* < 0.05). Since the data were not normally distributed, the median and interquartile range (IQR) were chosen to describe the data and their distributions. The variability of each measured parameter was quantified across the detected strides during each trial using the interquartile range (IQR), defined as the difference between the 75th and 25th percentiles of the distribution of stride-by-stride values. Strides were segmented based on consistent temporal markers extracted from the IMU signals. The IQR was calculated separately for each saddle condition and used as an indicator of the consistency of horse–rider movement patterns.

## 3. Results

Average horse speed was measured for each condition to verify its overall stability. Observed values showed little variation between saddles, allowing speed to be considered essentially constant in this study (Table S1).

### 3.1. Active Range of Motion (AROM) Protraction/Retraction (P/R) Fore and Hindlimbs

The AROM of the forelimbs varied significantly among saddles for all horses (Table 1). Compared to saddle A, saddle B had lower values for P1 (median [IQR]: AROM_B_ = 91.8° [4.6°] vs. AROM_A_ = 93.1° [4.3°], *p* = 0.002) and P6 (AROM_B_ = 85.2° [2.7] vs. AROM_A_ = 86.7° [2.5], *p* < 0.001). Similarly, saddles C and D showed significant differences for horses P2 and P6. For example, saddle C reduced P2’s AROM to 87.6° [4.3°], compared to 89.5° [6.2°] with saddle A (*p* < 0.001). Saddles B and C frequently have lower values than saddle A (such as P1 and P2). Variations in interquartile ranges (IQRs) were also observed across conditions. For instance, the IQR decreased with saddle C for P1 and P2. For P3, the IQR decreased with saddle B. For P4 and P5, IQR values remained similar across conditions. For P6, the IQR increased with saddles C and D. For P7, the IQR decreased with saddle C. For P8, the IQR increased with saddle D. Table 1 lists the horse-specific data, while Table 2 shows the rider-dependent parameters.

For hindlimbs AROM, each horse showed at least one significant variation between saddles B, C, and D with saddle A (Table 1). Saddle C often had significantly different values of saddle A, especially for P2 (AROM_C_ = 64.5° [3.5] vs. AROM_A_ = 66.6° [3.6°], *p* < 0.001) and P7 (AROM_C_ = 63.4° [3.5°] vs. AROM_A_ = 61.8° [4.1°], *p* < 0.001). Saddle D exhibited changes for P8 (AROM_D_ = 57.4° [2.9°] vs. AROM_A_ = 59.9° [2.1°], *p* < 0.001). Saddles C frequently have lower retraction AROM than saddle A, as demonstrated in P1, P2 and P4. This could indicate an impact on the horse’s hindlimbs propulsion. Variations in interquartile ranges (IQRs) were also noted. For P1, P2, and P5, IQRs remained similar across conditions. For P3, the IQR increased with saddle C. For P4, the IQR decreased with saddle D. For P6, the IQR decreased with saddles C and D. For P7, the IQR decreased with saddles C and D. For P8, the IQR increased with saddle D (Table 1).

### 3.2. Stride Time (ST)

**Figure 5:**
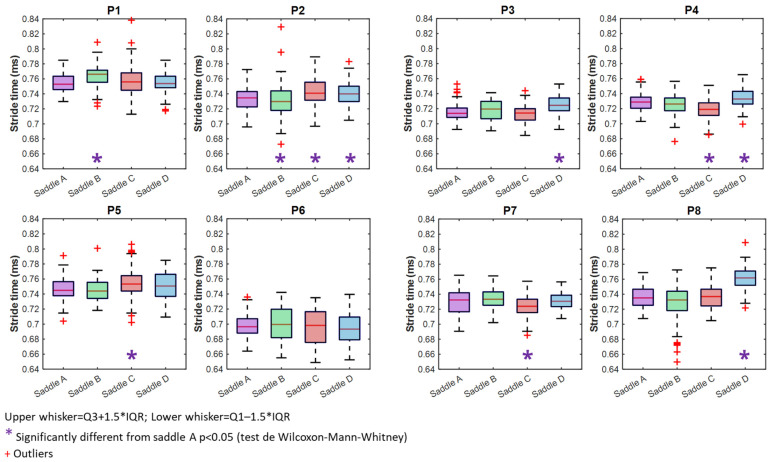
Boxplots illustrating stride time with saddles A (purple), B (green) C (red), and D (blue).

Except for P6, all horses had significantly longer stride times when wearing certain saddles (Figure 5) (Table 1). For P1 (ST_B_ = 765 ms [18.7 ms] vs. ST_A_ = 754 ms [17.8 ms], *p* < 0.001), saddle B resulted in a longer stride time than saddle A, and for P2, it is saddle C that resulted in longer stride time (ST_C_ = 741 ms [16.9 ms] vs. ST_A_ = 734 ms [19.6 ms], *p* < 0.001) (Figure 5). Saddle D significantly increased P8’s stride time (ST_D_ = 763 ms [20.7 ms] vs. ST_A_ = 736 ms [21.3 ms], *p* < 0.001). These increases may influence cadence or affect stride stability. Saddle C, and to a lesser extent, saddle D, had a longer stride time than saddle A (P2, P5, and P8). Interquartile ranges (IQRs) varied among horses. For P1, the IQR increased with saddle C, despite similar median values and no significant difference. For P2, the IQR increased with saddles C and D. For P3 and P5, the IQR increased with stride time for saddle D and C, respectively. For P4, the IQR decreased with saddle C and also decreased with saddle D, despite a longer stride time. For P6, no significant differences were observed. For P7 and P8, the IQR decreased with saddle C and D, respectively (Table 1).

### 3.3. Rider’s Pelvic AROM

For the majority of riders, pelvic pitch AROM differed significantly depending on the saddle (Table 2). Only P2 and P7 had substantial differences in AROM between saddles. Several riders have significantly reduced pelvic AROM when using saddles B, C, and D compared to saddle A. For P1, saddles B, C, and D show a decrease in pelvic pitch AROM (all *p* < 0.001) compared to saddle A, indicating a potential restriction in the rider’s pelvic mobility. P3 has considerably decreased pelvic pitch AROM with saddles B (*p* = 0.004) and D (*p* < 0.001) compared to saddle A. Riders P5, P6, and P8’s results, on the other hand, show that the three saddles increase pelvic pitch AROM when compared to saddle A. For example, P6 has less pelvic AROM with saddle A than the other three. Significant differences exist between saddles B (AROM_B_ = 6.9° [3.8°] vs. AROM_A_ = 6.0° [1.5°], *p* < 0.001) and C (AROM_C_ = 6.9° [1.6°] vs. AROM_A_ = 6.0° [1.5°], *p* < 0.001). We also observed a larger IQR with saddle B for the same rider, as well as P5 with saddle D. Only intra-pair comparisons were performed, although it is worth noting that some riders had a larger AROM than others. We see roughly a 10° change in amplitude between P2 and P7.

### 3.4. Rider–Horse Synchronization (Time Lag)

**Figure 6:**
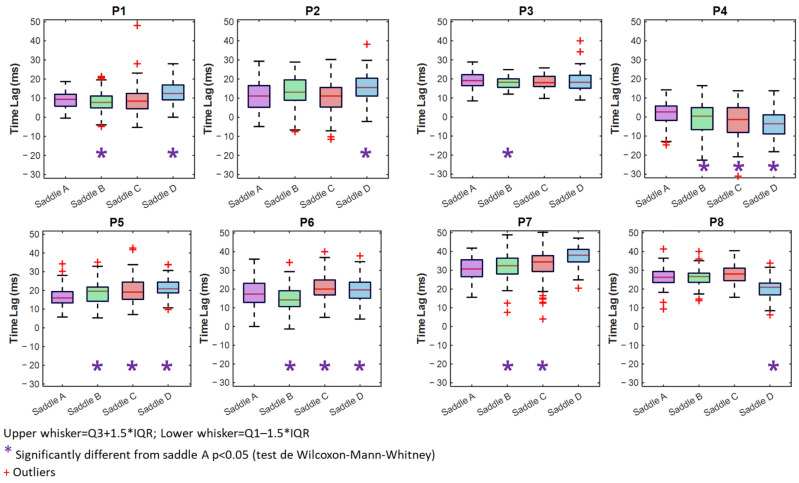
Boxplots illustrating Time Lag with saddles A (purple), B (green) C (red), and D (blue).

Time Lag values close to zero were interpreted as minimal phase delay between horse and rider movements; however, this does not imply a universally optimal synchronization, as the mechanical properties of the system can introduce inherent delays. All pairings exhibit significant changes based on the saddle. Except for P3 and P8, all pairs show a TL that is much greater than zero with saddle D than saddle A (Figure 6) (Table 2). For the pairs P4, P5, and P6, we can see that the TL is notably different from saddle A. For the pairs P4, P5, and P6, we can see that the TL is notably different from saddle A. For P4, for example, the TL is positive and closer to zero with the saddle B than saddle A, but the IQR is bigger with saddle B (TL_B_ = 0.4 ms [11.6 ms] vs. TL_A_ = 2.7 ms [7.6 ms], *p* = 0.015). Saddles C and D have negative TLs, with saddle C closer to 0 (TL_C_ = −1.3 ms [13.0 ms] vs. TL_A_ = 2.67 ms [7.6 ms], *p* < 0.001) and saddle D further from 0 (TL_D_ = −3.6 ms [10.0 ms] vs. TL_A_ = 2.7 ms [7.6 ms], *p* < 0.001). P4 has the lowest IQR with saddle A among the three saddles.

## 4. Discussion

The methodologies employed in this study demonstrate that IMUs enable the quantification of biomechanical changes in both the rider and the horse, as well as their synchronizations. Significant differences were found across all investigated parameters, highlighting the measurable influence of saddle design on equine biomechanics. This is particularly evident in limb AROM and stride time, supporting previous findings by Mackechnie-Guire et al. [26], who established that saddle characteristics directly influence freedom of movement and gait mechanics.

Our results reveal significant differences in the AROM of the forelimbs across the tested saddles, emphasizing the role of saddle design in limb mobility. These findings align with those of Murray et al. [27], who reported that pressure distribution from the saddle affects limb kinematics. This suggests that certain saddle adjustments may enhance forelimb AROM while others may restrict it, potentially due to variations in weight distribution and pressure on the thoracolumbar region.

Similarly, hindlimb AROM exhibited significant variations across more than half of the conditions tested. The impact of saddle fitting on hindlimbs kinematics has been previously highlighted by Greve & Dyson [28], who demonstrated that saddle fit influences spinal mobility and, consequently, limb movement patterns. Interestingly, our findings suggest that saddle fitting exerts a more pronounced effect on hindlimb AROM compared to the forelimbs. This specific disparity has not been extensively documented in the literature, although Murray et al. [27] reported that reduced pressure saddles facilitate greater protraction, likely due to increased spinal flexibility and modified muscular engagement, supporting better hindlimb function.

Given that saddle fitting influences limb AROM, it is expected that stride time would also be affected. Indeed, our study found significant differences in stride duration across seven of the eight rider–horse pairs. This supports the concept that saddle-induced changes in movement mechanics extend beyond localized limbs effects to broader locomotor dynamics.

Six out of the eight measured riders exhibited significant variations in pelvic AROM depending on the saddle used. A decrease in pelvic AROM suggests potential restrictions in postural mobility, whereas an increase may indicate either improved flexibility or reduced stability. This aligns with the findings of Wilkins et al. [29], who demonstrated that riders exhibit individual movement patterns, necessitating personalized analysis. Additionally, Persson-Sjodin et al. [30] highlighted that rider asymmetry can exacerbate asymmetry in the horse, a factor not directly measured in this study but relevant for future investigations. Moreover, previous studies [31] emphasize the role of pelvic tilt control in rider–horse synchronization, reinforcing the necessity of considering rider biomechanics when evaluating saddle fit.

Furthermore, gender-based differences in pelvic mobility have been noted in equestrian biomechanics research [32,33]. The literature indicates that male and female riders may exhibit distinct postural adaptations, potentially influencing stability and synchronization with the horse. While this study did not explicitly examine gender differences, future research could integrate this variable to further refine saddle fitting recommendations.

Time Lag, a crucial parameter reflecting the synchronization between horse and rider, exhibited significant differences across 16 conditions. In half of the cases where the TL shows a significant change in the rider–horse synchronization, this change is associated with a significant modification in the rider’s AROM and the horse’s AROM. These variations indicate that saddle modifications influence rider stability, postural adaptations, and the transmission of aids. Greve & Dyson [34] previously reported that saddle fit directly affects the rider’s capacity to follow the horse’s movements. Peham et al. [12] further established that reduced TL corresponds to enhanced harmony, suggesting that saddles promoting better support and mobility may enhance coordination. Conversely, an increased TL might reflect a delayed rider response due to altered weight distribution and balance.

Beyond the significance of average values, the analysis of variability—assessed via the interquartile range (IQR)—offers additional insights into the consistency of movement patterns. Across all parameters, some saddles induced larger IQRs, reflecting greater variability and potential instability, while others promoted more consistent movement. Notably, certain riders, such as P5 and P6, showed increased AROM or TL values accompanied by larger IQRs with certain saddles, suggesting a trade-off between amplitude and control. Conversely, other pairs maintained high TL values but low IQRs, such as P7, indicating that delayed coordination does not necessarily equate to irregular performance. These results emphasize that saddle effects are not limited to absolute changes in biomechanics but also affect movement consistency, a key aspect of effective and harmonious riding. Incorporating both central tendency and variability measures allows for a more nuanced evaluation of saddle–rider–horse synchronization and supports the need for individualized saddle-fitting strategies that account for both mobility and stability.

While this study provides valuable insights into the biomechanical effects of saddle fitting, several limitations should be noted. The small sample size and uncontrolled individual rider–horse characteristics may have influenced the findings. However, this limitation is partly mitigated by the large number of strides analyzed per condition (an average of 110, a minimum of 80), which enhances the robustness of the biomechanical measurements. Moreover, small sample sizes are common in equine science due to practical constraints [1,3,26,30]. Only four saddle modifications were tested, whereas other parameters—such as flap position, padding, or girth type—could also affect biomechanics. The same saddles were used across all horse–rider pairs, which did not ensure optimal individual fitting but allowed standardized comparisons. While our results highlight measurable biomechanical differences between saddle conditions, the study did not aim to identify the best-fitting saddle. Given that the same four saddles were used for all pairs, individual optimal fit was not guaranteed. Therefore, we cannot recommend one saddle as the “best” among those tested, and future studies should focus on personalized saddle fitting to establish criteria tailored to each horse–rider dyad.

Future studies using individually optimized saddles could better clarify the relationship between optimal saddle fit and biomechanical responses. Additionally, while IMU-derived biomechanical data objectively characterize rider–horse pair dynamics under different saddle conditions, their interpretation for determining optimal saddle fit relies on the expertise of qualified professionals. The data should therefore be considered a complementary tool rather than a standalone diagnostic method. Furthermore, although IMUs have been validated for equine and human biomechanical measurements, this study did not directly compare IMUs with a gold standard such as motion capture or pressure mapping. However, previous research has shown good agreement between IMUs and motion capture systems for both horse and rider kinematics [14,15,16,17], supporting the credibility of IMUs as an alternative method for biomechanical assessment. Nevertheless, future studies should include simultaneous recordings with reference systems to further validate IMUs in the specific context of saddle fitting.

This study focused on trot; future investigations at canter could provide further insights into gait-dependent effects of saddle modifications. Although a research-grade setup was used, the results highlight key parameters that could help develop more practical assessment tools with fewer sensors for routine saddle fitting evaluations. Lastly, although every effort was made to maintain a steady speed throughout data collection, small variations were observed across trials. Nevertheless, within each horse–rider pair, speed can be considered consistent across conditions. These minor fluctuations reflect the inherent variability of working in real-world field conditions and are representative of the natural dynamics of horse–rider interactions. Further research should explore a broader range of fitting criteria across larger and more diverse populations of horses and riders. Including a control saddle fitted to both horse and rider would also enhance data interpretation. Finally, the development of advanced algorithms to streamline data analysis would facilitate the real-world application of IMU-based assessments in equestrian biomechanics.

## 5. Conclusions

This study is conducted in an environment where saddle fitting is still largely based on subjective and empirical assessments. The lack of objective and easily applied tools is an important constraint in optimizing the saddle–rider–horse connection. Our findings represent a significant advancement by demonstrating that inertial sensors provide a credible alternative for assessing the impact of fitting, perhaps leading to a more scientific and standardized approach to saddle fitting. This study demonstrates that even minor saddle fitting modifications significantly affect the biomechanics of both horse and rider. These effects can be quantified using IMUs, providing an objective and reproducible method for evaluating saddle fit. Our findings underscore the necessity of precise saddle adjustments to optimize equine and rider performance and welfare. The integration of inertial sensors into saddle fitting assessments marks a promising step toward a more evidence-based approach in equestrian sports.

## Figures and Tables

**Table 1:** Horse’s biomechanical parameters.

		SADDLE A		SADDLE B			SADDLE C			SADDLE D		
		Med	IQR	Med	IQR	*p*-Value	Med	IQR	*p*-Value	Med	IQR	*p*-Value
**P1**	AROM P/R forelimbs (°)	93.1	4.3	91.8 *	4.6	0.002	91.1 *	3.5	<0.001	92.5	4.3	0.088
	AROM P/R hindlimbs (°)	63.3	4.0	62.1 *	3.7	<0.001	62.3 *	3.8	<0.001	63.4	3.7	0.73
	Stride time (ms)	754	17.8	765 *	18.7	<0.001	756	24.9	0.437	755	19.1	0.478
**P2**	AROM P/R forelimbs (°)	89.5	6.2	89.2	6.0	0.751	87.6 *	4.3	<0.001	87.8 *	4.9	0.004
	AROM P/R hindlimbs (°)	66.6	3.6	66.1	3.1	0.171	64.5 *	3.5	<0.001	66.2	3.2	0.167
	Stride time (ms)	734	19.6	729 *	21.3	0.035	741 *	16.9	<0.001	738 *	15.9	0.003
**P3**	AROM P/R forelimbs (°)	91.9	4.0	93.5 *	3.4	0.001	93.5	3.7	0.068	92.0	4.0	0.142
	AROM P/R hindlimbs (°)	65.8	3.4	67.3 *	3.6	<0.001	66.6 *	4.2	<0.001	66.0	4.1	0.310
	Stride time (ms)	714	12.4	717	24.4	0.124	713	16.9	0.412	724 *	18.7	<0.001
**P4**	AROM P/R forelimbs (°)	83.7	3.2	83.8	3.3	0.888	84.2	4.0	0.506	82.8 *	3.7	0.002
	AROM P/R hindlimbs (°)	63.5	4.2	63.2	3.2	0.125	62.6 *	4.4	0.008	62.1 *	3.5	<0.001
	Stride time (ms)	729	17.6	727	18.7	0.104	720 *	15.8	<0.001	733 *	16.9	0.006
**P5**	AROM P/R forelimbs (°)	87.8	4.0	89.1 *	4.4	0.005	87.9	3.6	0.300	87.5	4.3	0.261
	AROM P/R hindlimbs (°)	63.4	4.0	64.7 *	4.1	0.002	64.1 *	3.8	0.046	63.2	3.8	0.589
	Stride time (ms)	745	19.8	744	22.2	0.540	753 *	23.1	<0.001	751	29.8	0.086
**P6**	AROM P/R forelimbs (°)	86.7	2.5	85.2 *	2.7	<0.001	87.6 *	3.5	0.006	87.8 *	3.1	<0.001
	AROM P/R hindlimbs (°)	56.3	3.8	55.0 *	3.3	<0.001	57.4 *	1.8	0.002	57.0 *	2.9	0.003
	Stride time (ms)	696	18.7	700	35.6	0.262	696	39.9	0.324	692	26.9	0.110
**P7**	AROM P/R forelimbs (°)	91.2	3.4	91.1	2.6	0.617	92.0 *	2.4	0.005	90.6	3.4	0.447
	AROM P/R hindlimbs (°)	61.8	4.0	62.3	3.7	0.537	63.4 *	3.5	<0.001	63.1 *	3.6	0.008
	Stride time (ms)	733	23.1	734	16.0	0.247	725 *	17.8	<0.001	731	12.4	0.556
**P8**	AROM P/R forelimbs (°)	88.9	3.6	89.0	3.6	0.302	89.0	3.1	0.476	87.0 *	4.1	<0.001
	AROM P/R hindlimbs (°)	59.9	2.1	60.2	2.6	0.174	59.6	2.5	0.226	57.4 *	2.9	<0.001
	Stride time (ms)	736	21.3	733	24.0	0.100	737	20.4	0.545	763 *	20.7	<0.001

P: rider–horse pair; med: median; IQR: interquartile range; no *p*-value for saddle A as it is our control saddle. * Significantly different from saddle A *p* < 0.05 (Wilcoxon–Mann–Whitney).

**Table 2:** Riders’ specific parameters.

		SADDLE A		SADDLE B			SADDLE C			SADDLE D		
		Med	IQR	Med	IQR	*p*-Value	Med	IQR	*p*-Value	Med	IQR	*p*-Value
**P1**	Pelvic pitch AROM (°)	8.9	1.3	8.5 *	1.4	<0.001	7.9 *	1.2	<0.001	7.3 *	1.4	<0.001
	TL (ms)	9.3	6.2	7.8 *	6.2	0.048	8.4	8.0	0.296	12.4 *	7.8	<0.001
**P2**	Pelvic pitch AROM (°)	3.5	1.6	3.8	1.2	0.326	3.7	1.0	0.425	3.7	1.3	0.304
	TL (ms)	11.1	11.3	13.1	10.7	0.054	11.1	10.2	0.599	15.6 *	9.3	<0.001
**P3**	Pelvic pitch AROM (°)	6.1	1.1	5.8 *	0.9	0.004	5.9	0.7	0.116	5.4 *	1.0	<0.001
	TL (ms)	19.1	5.8	18.2 *	4.4	0.010	18.0	5.3	0.082	18.2	6.8	0.181
**P4**	Pelvic pitch AROM (°)	5.0	1.0	5.0	1.1	0.408	4.6 *	0.9	0.001	5.6 *	1.2	<0.001
	TL (ms)	2.7	7.6	0.4 *	11.6	0.015	−1.3 *	13.0	<0.001	−3.6 *	10.0	<0.001
**P5**	Pelvic pitch AROM (°)	3.8	1.1	4.4 *	1.8	<0.001	3.9	1.4	0.081	6.2 *	6.0	<0.001
	TL (ms)	16.0	6.0	19.6 *	7.6	0.009	19.1 *	9.2	<0.001	20.9 *	5.8	<0.001
**P6**	Pelvic pitch AROM (°)	6.0	1.5	6.9 *	3.8	<0.001	6.9 *	1.6	<0.001	6.3	1.2	0.060
	TL (ms)	17.3	10.2	14.2 *	8.4	<0.001	20.0 *	8.0	0.001	19.6 *	8.6	0.045
**P7**	Pelvic pitch AROM (°)	12.2	7.1	11.8	1.8	0.716	12.8	7.4	0.585	12.0	3.4	0.635
	TL (ms)	30.7	9.0	32.4	8.4	0.107	34.4 *	8.4	0.017	38.0 *	6.7	<0.001
**P8**	Pelvic pitch AROM (°)	4.9	1.1	5.1	1.4	0.659	5.1	1.1	0.126	5.5 *	0.9	<0.001
	TL (ms)	26.2	5.9	26.7	4.9	0.826	28.0	6.7	0.126	20.9 *	6.2	<0.001

P: rider–horse pair; med: median; IQR: interquartile range; no *p*-value for saddle A as it is our control saddle. * Significantly different from saddle A *p* < 0.05 (Wilcoxon–Mann–Whitney).

## Data Availability

Data is contained within the article.

## Supplementary Materials

The following supporting information can be downloaded at https://www.mdpi.com/article/10.3390/s25154712/s1: Table S1: Average trotting speed (km/h ± SD) for each rider-horse pair and saddle condition.
