# Supplementary material for: Quantification of the Effect of Saddle Fitting on Rider–Horse Biomechanics Using Inertial Measurement Units
Source: Sensors (Basel). 2025 Jul 30;25(15):4712. doi: 10.3390/s25154712 (PMC12349058; doi:10.3390/s25154712)
Supplement: Supplementary file 1 [file sensors-25-04712-s001.zip › sensors-3734980-supplementary.pdf]

sensors

MDPI

Supplementary material

Table S1. Average trotting speed (km/h ± SD) for each rider-horse pair and saddle condition.

	Saddle A	Saddle B	Saddle C	Saddle D
P1	12.69 ± 0.33	12.38 ± 0.38	12.50 ± 0.44	12.95 ± 0.22
P2	13.21 ± 0.33	13.07 ± 0.22	12.91 ± 0.37	12.99 ± 0.41
P3	13.45 ± 0.38	12.99 ± 0.41	13.19 ± 0.65	13.04 ± 0.29
P4	12.87 ± 0.42	12.70 ± 0.30	13.01 ± 0.40	12.78 ± 0.17
P5	14.31 ± 0.34	14.13 ± 0.29	13.70 ± 0.28	13.46 ± 0.45
P6	12.68 ± 0.28	12.26 ± 0.56	13.05 ± 0.48	12.85 ± 0.56
P7	13.27 ± 0.29	13.26 ± 0.60	13.68 ± 0.27	13.42 ± 0.60
P8	12.70 ± 0.07	12.96 ± 0.19	12.78 ± 0.12	12.26 ± 0.36

P: rider-horse pair
